# Additional Impact of Glucose Tolerance on Telomere Length in Persons With and Without Metabolic Syndrome in the Elderly Ukraine Population

**DOI:** 10.3389/fendo.2019.00128

**Published:** 2019-02-28

**Authors:** Mykola D. Khalangot, Dmytro S. Krasnienkov, Valentina P. Chizhova, Oleg V. Korkushko, Valery B. Shatilo, Vitaly M. Kukharsky, Victor I. Kravchenko, Volodymyr A. Kovtun, Vitaly G. Guryanov, Alexander M. Vaiserman

**Affiliations:** ^1^Epidemiology Department, Komisarenko Institute of Endocrinology and Metabolism, Kyiv, Ukraine; ^2^Endocrinology Department, Shupyk National Medical Academy of Postgraduate Education, Kyiv, Ukraine; ^3^Laboratory of Epigenetics, Chebotariov Institute of Gerontology, Kyiv, Ukraine; ^4^Public Health Management Department, Bogomolets National Medical University, Kyiv, Ukraine

**Keywords:** metabolic syndrome, telomeres, impaired glucose tolerance, artificial neural networks, Ukraine

## Abstract

**Rationale:** Association between different components of metabolic syndrome and the rate of age-related telomere shortening was reported repeatedly, although some findings are inconsistent across studies, suggesting the need for further research on the topic. In the present study, we examined relationships between different components of metabolic syndrome (MetS); glucose tolerance reflected in 2-h post-load plasma glucose (2hPG) levels and age on the leukocyte telomere length (LTL) in Ukraine population.

**Methods:** The study was conducted on the 115 adult individuals residing in the Kyiv region (Ukraine). Among them, 79 were diagnosed with MetS according to the International Diabetes Federation definition. LTL were determined by a qPCR-based method. Multivariate logistic regression (MLR) and artificial neural networks (ANN) modeling were used for the analysis of the results. ROC-analysis was also performed to compare the predictively values of this models.

**Results:** MetS was associated with a high (OR = 3.0 CI 1.3–6.7; *p* = 0.01) risk of having shorter telomeres that remained significant after adjusting for age, gender and 2hPG levels. Fasting plasma glucose (FPG) levels and other MetS components did not affect the magnitude of the relationship and did not reveal the independent influence of these factors. The level of 2hPG in turn, demonstrated a significant relationship (OR = 1.3 CI 1.0–1.6 per 1 mmol/l; *p* = 0.04) with LTL regardless of the presence of MetS. The non-linearity of the interactions between age, gender and 2hPG level was revealed by neural network modeling (AUC = 0.76 CI 0.68–0.84).

**Conclusion:** Our study found that impaired glucose tolerance, but not FPG levels, affected the association between LTL and MetS, which may be also indicative for pathophysiological differences in these hyperglycemia categories. 2hPG levels can provide an opportunity for a more accurate diagnostics of MetS and for evaluating the rate of aging in patients with MetS. Further research, however, is needed to verify this assumption.

Metabolic syndrome (MetS) is defined as a cluster condition of cardiovascular risk factors, including abdominal obesity, impaired fasting plasma glucose (FPG), hypertension, as well as low high-density lipoprotein (HDL) and/or high triglyceride levels (1). MetS was repeatedly found to be associated with many signs of unhealthy aging, such as circadian rhythm disturbances, sarcopenic obesity, loss of muscle mass, ectopic fat accumulation, insulin resistance, impaired magnesium metabolism, systemic inflammation (2), depression (3), and dementia (4) in older individuals. MetS was also shown to be associated with accelerated telomere shortening with age (5). Telomeres are DNA-protein complexes at the end of eukaryotic chromosomes that cap ends of chromosomes and protect the contained genetic material to promote chromosomal stability (6). The telomere lengths (TLs) are regulated by telomerase, an RNA-dependent DNA polymerase complex that catalyzes the addition of telomeric repeats (TTAGGG) to the ends of chromosomes to compensate for the end-replication problem. In most somatic cells, telomeres shorten throughout successive cell divisions (a process commonly referred to as “telomere attrition”) in consequence of insufficient telomerase activity. Therefore, cellular replication may be carried out until a critical threshold of TL is reached (7). Therefore, a rate of telomere shortening is regarded as an indicator of replicative senescence and leukocyte TL (LTL) is currently widely applied as a biomarker of aging (8, 9). Telomeres, owing to their chemical structure, are highly susceptible to oxidative damages (10). Since MetS is known to induce chronic oxidative stress and related inflammation (11), it is not surprising that trends to decreased telomerase activity and accelerated telomere attrition have been repeatedly found in patients with MetS. For example, in research by Révész et al. (12), shortened LTLs predicted risk of each of the components of MetS, except for hypertension. Significant association between different components of metabolic syndrome and the rate of age-related telomere shortening was reported repeatedly (12–17). This association, however, was not consistent in several studies (18, 19). This inconsistency suggests that further studies are needed to better understand mechanisms underlying these associations. In Ukraine, these associations have never been examined comprehensively until now. An inverse relationship between LTL and age was shown in Ukrainians with normal FPG levels, while no such association was found in persons with impaired glucose metabolism reflected in abnormal FPG levels (20). Moreover, significant inverse association between LTL and 2-h post-load plasma glucose (2hPG) levels has been revealed in our previous study (21). In the present study, we aimed to further examine this relationship in patients with and without MetS.

## Methods

### Participants

One hundred and fifteen (29% of men) residents of Kyiv region of Ukraine were examined between 2013 and 2016. Among them, 34 persons were recruited from the clinic of Institute of Gerontology (Kyiv, Ukraine) and 81 persons were recruited under the supervision of the Institute of Endocrinology and Metabolism from two local family medicine clinics of the Makariv rural district (Kyiv region). The inclusion criteria were as follows: (1) middle-to-old age persons (43–87 years old); (2) lack of previously diagnosed T2D. The exclusion criteria were as follows: (1) residence in a region other than Kyiv; (2) inability to visit the clinic due to either chronic illness or disability; (3) refusal or inability to give informed consent. 79 (68.7%) of persons studied had metabolic syndrome according to the current International Diabetes Federation (IDF) definition (1).

### Ethical Aspects

The study protocol was approved by the Ethics Committees of the Institute of Endocrinology and Metabolism and Institute of Gerontology (both are part of the National Academy of Medical Sciences of Ukraine). All participants provided written informed consent. The Declaration of Helsinki (2000) and the applicable national standards as they relate to the involvement of human subjects in research were enforced.

### Collection and Storage of Blood Samples

For the FPG test, blood was collected from all volunteers after a 12 h fast. For the oral glucose tolerance test (OGTT), blood was collected 2 h after ingestion of glucose (75 g of glucose per 200 mL of water) in the morning after at least 10 h of fasting. Blood was collected in EDTA-coated tubes and centrifuged at 1,000 g for 10 min. For DNA extraction, blood was collected in EDTA-coated tubes and stored at −80°C until DNA extraction procedure.

### Measurement of Baseline Characteristics

BMI was determined as the body weight (kg) divided by the height (m) squared (kg/m^2^). Waist circumference (WC) was measured at the point of noticeable waist narrowing using a flexible anthropometric tape, with the subject in a standing position. Hip circumference (HC) was measured at the maximum circumference over the buttocks. The waist-hip ratio (WHR) was calculated as the ratio between WC and HC. Systolic blood pressure (Systolic BP) and diastolic blood pressure (Diastolic BP) (mm Hg) were measured twice with a standard sphygmomanometer in a sitting position after at least 10 min of rest. Plasma glucose levels were determined by a standard glucose oxidase method. HDL cholesterol and triglycerides plasma levels were determined by using the automatic analyzer GBG ChemWell 2910 (Awareness Tech. Inc., USA) and corresponding kits (Global Scientific T5532-1000 and H-7545-320). Since LTL levels were shown to be significantly associated with 2hPG levels in our previous research (21), 2hPG data were also included in present study even though this metabolic indicator is currently commonly used as diagnostic criteria for pre-diabetes and Type 2 diabetes but not in the diagnosis of MetS (1).

### Telomere Length Assay

The relative telomere lengths (RTLs) were measured by monochrome multiplex polymerase chain reaction in real time (qPCR) following the method described by Cawthon (22). DNA was extracted from the whole blood using the phenol-chloroform purification method (23). PCR reaction mix was prepared using a commercial reagent kit for RT-PCR (2.5x Reaction mix for the RT-PCR with SYBR Green I, M-427, Syntol, Russian Federation) with addition of betaine (B0300-1VL, Sigma-Aldrich, USA) at a final concentration of 1M. For multiplex qPCR, the telomere primer pair telg and telc (final concentrations 450 nM each) were combined with the albumin primer pair albu and albd (final concentrations 250 nM each) in the master mix. The thermal cycling profile was as follows: 15 min at 95°C; 2 cycles of 15 s at 94°C, 15 s at 49°C; and 32 cycles of 15 s at 94°C, 10 s at 62°C, 15 s at 74°C with signal acquisition, 10 s at 84°C, and 15 s at 88°C with signal acquisition. To obtain the calibration curve, PCR was carried out at four concentrations of the reference DNA in duplicates which cover a range of 27-fold dilutions, prepared by serial dilution. All DNA samples were run in triplicate. Amplification curves were generated by the Opticon Monitor 3 software. After the thermal cycling and raw data collection was completed, the Opticon Monitor 3 software was used to generate two standard curves for each plate, one for the telomere signal and another for the single-copy gene (scg) albumin signal. The telomere length was expressed as the T/S ratio, the telomere repeat copy number (T) to the scg copy number (S).

### Statistical Analysis

The Shapiro-Wilk test was used to check the normality of variable distributions. For normally distributed data, the mean and standard deviation (SD), for non-normally distributed numerical data the median and inter quartile range (Q_I_-Q_III_) were calculated. Student *t*-test or the Mann-Whitney U-test for comparison of two independent samples has been used. Fisher's exact test was used to determine differences for nominal data. Multivariate logistic regression (MLR) models were used to estimate odds ratios (OR) and 95% confidence intervals (CIs) for the association between T/S ratio (dependent variable) and independent variables. Artificial neural networks (ANN) were used to estimate the non-linear association between T/S ratio and independent variables (multilayer perceptron MLP, with the logistic activation functions was created) (24). For comparison of the predictively values of logistic regression model (multiplicative model) and MLP (non-linear model), ROC-analysis was performed (Area Under Curves—AUC's, were compared). The significance threshold was set at *p* < 0.05. When building logistic models such categories were used: shorter LTL—T/S ratio < 0.703 (low and middle tertiles); longer LTL—T/S ratio above 0.703. FPG, 2hPG, WC, HDL cholesterol, triglycerides plasma levels and age are considered as continuous variables.

Analyses was performed by MedCalc v.18.10 (MedCalc Software Inc., Broekstraat, Belgium, 1993–2018). For analyses of ANN's models Statistica Neural Networks v. 4.0C (StatSoft Inc., USA, 1996–1999) was used.

## Results

Anthropometric and biochemical characteristics, and also LTL (T/S ratio) of the studied population are presented in Table 1.

**Table 1:** Baseline characteristics of study population.

**Characteristics**	**All (*n* = 115)**	**MetS free population (*n* = 36)**	**MetS population (*n* = 79)**	***p***
Age, years, Mean ± SD	63 ± 10.1	64.4 ± 11.5	62.4 ± 9.5	0.32^a^
Men, *n* (%)	33 (28.7)	17 (47.2)	16 (20.3)	0.006
T/S, Me (Q_I_-Q_III_)	0.61 (0.5–0.72)	0.71 (0.57–0.79)	0.59 (0.48–0.7)	0.012^b^
Waist circumference, cm, Mean ± SD	102.1 ± 14.6	91.3 ± 12.8	106.9 ± 12.7	< 0.001^a^
Hip circumference, cm, Me (Q_I_-Q_III_)	109 (101–118)	101 (97–105)	113 (107–122)	< 0.001^b^
Waist/Hip circumference, Mean ± SD	0.93 ± 0.08	0.89 ± 0.07	0.94 ± 0.07	0.002^a^
Neck circumference, cm, Me (Q_I_-Q_III_)	37 (35–39)	35.5 (33.5–38)	37 (36–40)	0.015^b^
Weight, kg, Mean ± SD	84.7 ± 17.4	73.4 ± 13.5	89.8 ± 16.6	< 0.001^a^
Height, cm, Me (Q_I_-Q_III_)	162 (158–167)	164 (158–172)	162 (158–1667)	0.383^b^
BMI, kg/m^2^, Me (Q_I_-Q_III_)	31.3 (27.7–35.9)	27.3 (23.7–29.3)	32.5 (30.2–37.6)	< 0.001^b^
FPG, mmol/l, Me (Q_I_-Q_III_)	5.98 (5.48–6.7)	5.39 (4.87–6.22)	6.04 (5.74–6.79)	< 0.001^b^
2hPG, mmol/l, Me (Q_I_-Q_III_)	6.4 (5.1–7.6)	6.2 (4.8–7.7)	6.4 (5.3–7.6)	0.46^b^
Systolic BP, mmHg, Me (Q_I_-Q_III_)	135 (125–155)	125 (115–137)	140 (127–160)	< 0.001^b^
Diastolic BP, mmHg, Me (Q_I_-Q_III_)	82 (80–95)	80 (77–86.5)	87 (80–95)	0.035^b^
Triglycerides, mmol/l, Me (Q_I_-Q_III_)	0.91 (0.69–1.22)	0.81 (0.58–1.03)	0.96 (0.72–1.25)	0.03^b^
HDL, mmol/l, Me (Q_I_-Q_III_)	1.22 (0.97–1.40)	1.38 (1.25–1.525)	1.12 (0.90–1.28)	< 0.001^b^
Smokers, *n* (%)	2 (1.8)	2 (6.1)	0 (0)	0.09^c^
Alcohol using, *n* (%)	52 (47.7)	13 (39.4)	37 (48.7)	0.41^c^

*Normally distributed characteristics are expressed as mean ± standard deviation (SD) values. Non-normally distributed characteristics are expressed as medians (Q_I_-Q_III_)*.

a *Means are compared by the Student t-test*.

b *Medians are compared by the Mann-Whitney U-test*.

c *Qualitative characteristics are compared by the Fisher's exact test*.

As we can see in the Table, persons with MetS were characterized by a significantly shorter TLs compared with those in subjects without MetS. In the population we studied, women prevailed, but in the MetS category there were significantly more women. Other anthropometric and biochemical characteristics studied differed in the expected manner between individuals with and without MetS, thereby confirming the correctness of the phenotypic categories identified (Table 1).

Association between LTL and the presence of MetS was estimated using logistic regression models (Tables 2, 3). Table 2 presents the results of one-factor regression model for predicting the risk of having shorter TLs (lower vs. middle and upper T/S tertiles) depending on the presence or absence of MetS, and also two-factor regression models adjusted for variables used in categorizing MetS, including FPG, waist circumference, HDL cholesterol, triglycerides, and systolic BP. Table 3 presents the results of two-factor regression model adjusted for variables non-used in categorizing MetS, such as age, gender, alcohol consumption, smoking, and also 2hPG.

**Table 2:** The effect of individual components of MetS on the LTL depending on the presence or absence of MetS.

**Independent variables**	**Regression coefficients, b ± m**	**Significance level, *p***	**OR (95% CI)**	**AUC (95% CI)**
**ONE-FACTOR MODEL**				
MetS	1.08 ± 0.42	**0.01**	3.0 (1.3–6.7)	0.62 (0.53–0.71)
**TWO-FACTOR MODEL, MetS** **+** **FPG**				
MetS	0.93 ± 0.45	**0.04**	2.5 (1.1–6.1)	0.68 (0.59–0.77)
FPG	0.19 ± 0.20	0.33	1.2 (0.8–1.8)	
**TWO-FACTOR MODEL, MetS** **+** **WAIST CIRCUMFERENCE**				
MetS	0.95 ± 0.50	0.054	2.6 (1.0–6.8)	0.63 (0.54–0.72)
Waist Circumference	0.005 ± 0.016	0.78	1.00 (0.97–1.04)	
**TWO-FACTOR MODEL, MetS** **+** **HDL CHOLESTEROL**				
MetS	1.07 ± 0.45	**0.02**	2.9 (1.2–7.1)	0.63 (0.53–0.71)
HDL cholesterol	−0.02 ± 0.68	0.98	1.0 (0.3–3.7)	
**TWO-FACTOR MODEL, MetS** **+** **TRIGLYCERIDES**				
MetS	1.01 ± 0.43	**0.02**	2.7 (1.2–6.4)	0.62 (0.52–0.71)
Triglycerides	0.26 ± 0.33	0.42	1.3 (0.7–2.5)	
**TWO-FACTOR MODEL, MetS** **+** **HIGH BP**				
MetS	1.06 ± 0.45	**0.02**	2.9 (1.2–6.9)	0.62 (0.53–0.71)
High BP	−0.08 ± 0.43	0.86	0.9 (0.4–2.2)	

*The significant values (p < 0.05) are marked with bold*.

**Table 3:** The risk of having shorter TLs depending on the presence or absence of MetS: the influence of factors that are not included in the definition of MetS.

**Independent variables**	**Regression coefficients, b, m**	**Significance level, *p***	**OR (95% CI)**	**AUC (95% CI)**
**ONE-FACTOR MODEL**				
MetS	1.08 ± 0.42	**0.01**	3.0 (1.3–6.7)	0.62 (0.53–0.71)
**TWO-FACTOR MODEL, MetS** **+** **AGE**				
MetS	1.18 ± 0.44	**0.007**	3.3 (1.4–7.7)	0.66 (0.56–0.74)
Age	0.036 ± 0.021	0.09	1.04 (0.99–1.08)	
**TWO-FACTOR MODEL, MetS** **+** **ALCOHOL INTAKE**				
MetS	0.87 ± 0.44	**0.049**	2.4 (1.0–5.6)	0.60 (0.51–0.70)
Alcohol using	−0.15 ± 0.42	0.73	0.9 (0.4–3.9)	
**TWO-FACTOR MODEL, MetS** **+** **GENDER**				
MetS	0.95 ± 0.44	**0.03**	2.6 (1.1–6.1)	0.64 (0.55–0.73)
Gender	−0.51 ± 0.45	0.26	0.6 (0.2–1.5)	
**TWO-FACTOR MODEL, MetS** **+** **2hPG**				
MetS	1.04 ± 0.43	**0.02**	2.8 (1.2–6.6)	0.68 (0.59–0.77)
2hPG	0.23 ± 0.11	**0.04**	1.3 (1.0–1.6)	
**FOUR-FACTOR MODEL, MetS** **+** **AGE** **+** **GENDER** **+** **2hPG**				
MetS	1.01 ± 0.46	**0.03**	2.7 (1.1–6.8)	0.69 (0.60–0.77)
Age	0.027 ± 0.022	0.22	1.03 (0.98–1.07)	
Gender	−0.47 ± 0.47	0.31	0.6 (0.3–1.6)	
2hPG	0.20 ± 0.11	0.06	1.2 (1.0–1.5)	

*The significant values (p < 0.05) are marked with bold*.

The presence of MetS was associated with high odds of having shorter telomeres: OR 3.0 (1.3–6.7), *p* = 0.01 (Table 2). The association was significant after adjusting for variables both applied (Table 2) and non-applied (Table 3) in categorizing MetS. By contrast, LTL was strongly associated with 2hPG level, independently of MetS presence, which is reflected in a slight increase of the area under the ROC-curve: AUC = 0.68 (0.59–0.77) vs. 0.62 (0.53–0.71) for the crude model (Table 3).

The use of 2-factor models to assess the impact of high FPG levels and other MetS components on the relationship between LTL and MetS did not affect the magnitude of the relationship and did not reveal the independent influence of these factors (Table 2). After adjusting for all variables non-used in categorizing MetS (age + gender + 2hPG), the risk of having short telomeres remained to be significant, although AUC increased only slightly: 0.69 (0.60–0.77).

**Figure 1:**
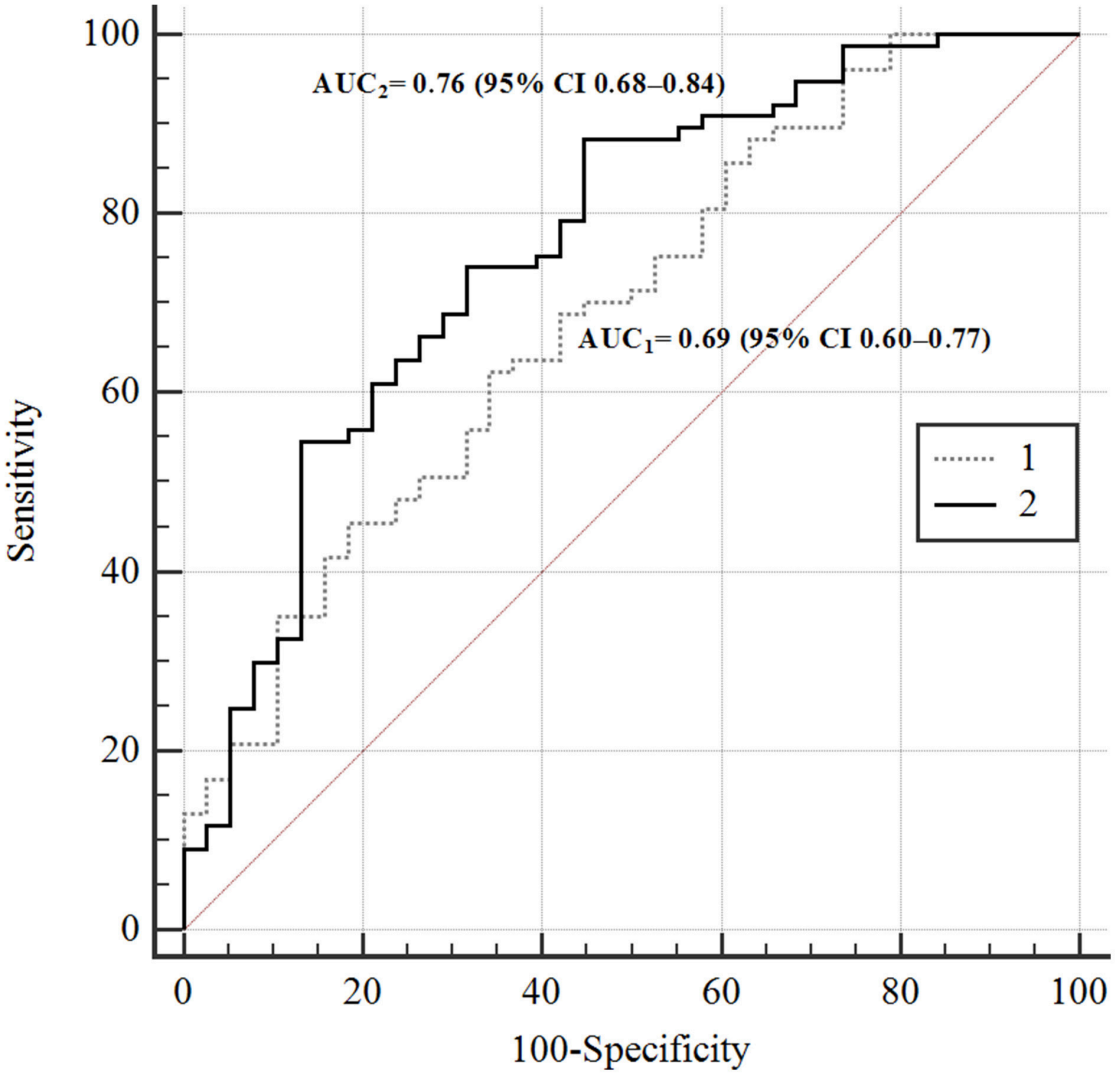
ROC-curves of the risks of TL's reducing according to different prediction models. In the figure: 1–4-th factorial logistic regression model; 2—ANN 4-th factorial neural network (MLP) prediction model. AUC_1_ = 0.69 (95% CI 0.60–0.77); AUC_2_ = 0.76 (95% CI 0.68–0.84).

To test the possibility of non-linear interactions between LTL, age, and metabolic characteristics, the feedforward neural network (MLP) model (24) was created. The model architecture is given in Figure S1. Figure 1 shows the prediction of the risk of reducing LTL according to this model (AUC_2_) in comparison with logistic regression model (AUC_1_).

**Figure 2:**
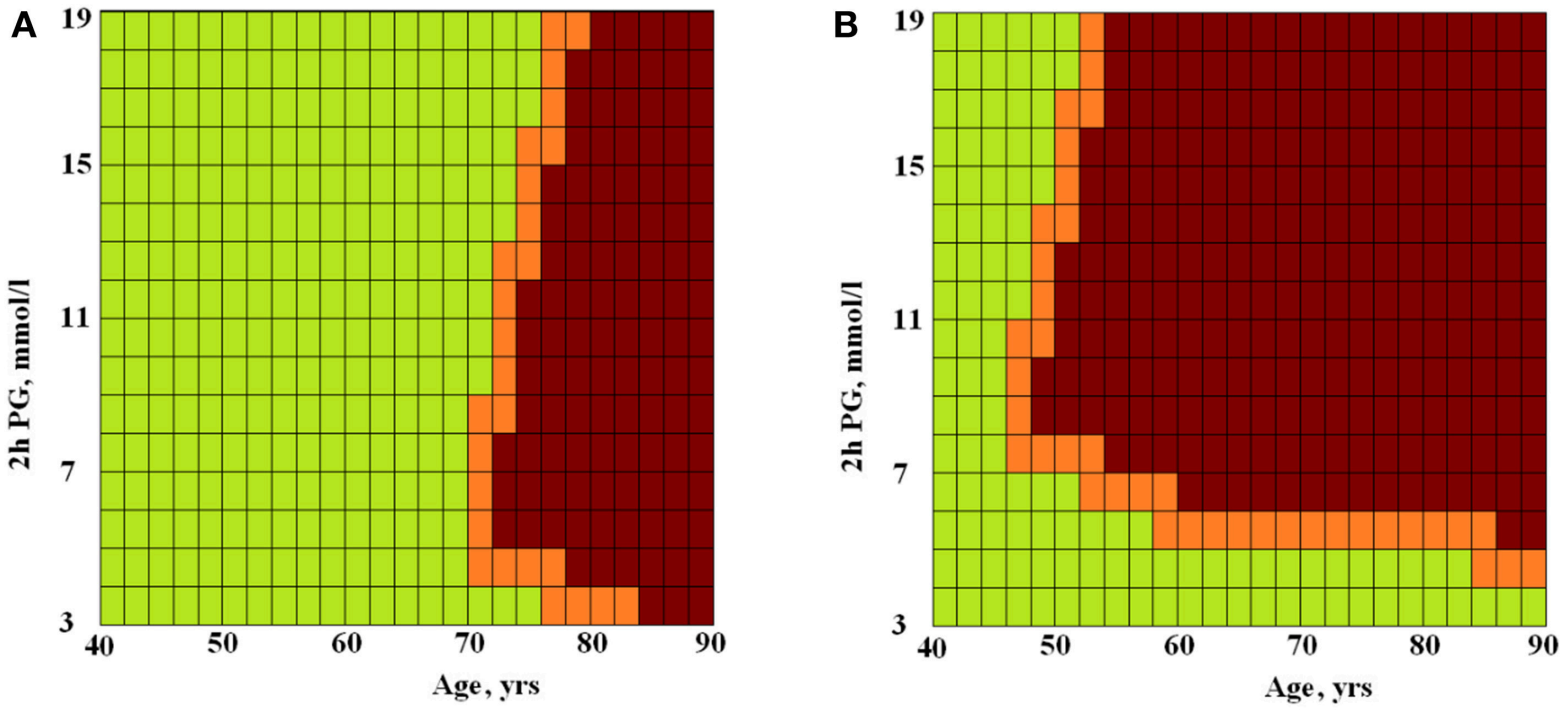
The ANN modeled risks of having longer or shorter TLs for persons without **(A)** and with **(B)** MetS depending on age and 2hPG level. Green squares, longer TLs; brown squares, shorter TLs; X-axis, age (years) and Y-axis, 2hPG (mmol/l).

The difference between the areas was statistically significant, *p* = 0.02. This is evidence of non-linarites. Figure 2 shows the risks of having longer (green squares) or shorter (brown squares) TLs for men and women depending on the presence or absence of MetS depending on age (X-axis) and 2hPG (Y-axis). The risk of telomere shortening in the presence of MetS is clearly visible. In addition, for persons without MetS, the intermediate line between shorter and longer telomeres passes almost vertically on the border of 70 and 76 years. Long telomeres were revealed even in 90 years-old with MetS in case they had low 2hPG levels. At higher levels of 2hPG (7 mmol/l), odds of having long telomeres persisted only up to the age of 46 years in persons with MetS. In persons without MetS, impaired glucose tolerance (an increase of 2hPG) has almost no effect on the risk of telomere shortening.

## Discussion

In our study, MetS was associated with a high (3-fold) risk of having shorter telomeres, and this risk remained significant after adjusting for age, gender, and 2hPG level. The level of 2hPG, in turn, demonstrated a significant relationship with TL regardless of the presence of MetS. The non-linearity of the interactions between TL, MetS, age, gender, and 2hPG level was revealed by neural network modeling (Figure 1). In Figure 2, this non-linearity is visually evident from the fact that, in the presence of MetS, the odds of having longer telomeres were associated with young age and/or high glucose tolerance (expressed by the low 2hPG level). Longer TLs were observed up to 90 years of age if 2hPG level did not exceed 4 mmol/l), and up to 46 years of age only if levels of 2hPG exceeded 7 mmol/l. In the absence of MetS, the risk of having short telomeres changed with age and was less dependent on glucose tolerance. To the best of our knowledge, the non-linearity of associations of TL with age, glucose tolerance, and MetS has not been previously described. Perhaps one explanation for this phenomenon may be an increase in telomerase activity in individuals with the MetS subtype, characterized by a strong dyslipidemic profile together with the lack of hyperglycemia (25). It can be assumed that telomerase activity could be increased in patients with MetS but without chronic hyperglycemia, thereby providing the possibility to have long TLs. Visualization of the chances of detecting long TL in individuals with MetS but with good glucose tolerance, regardless of age, was provided by ANN modeling in our study (see Figure 2).

Overall, associations found in this study may be discussed in the context of present-day debates about the difference between pathophysiological backgrounds in different hyperglycemia categories determined by FPG or 2hPG levels (26–29). Fasting hyperglycemia is known to be caused by elevated endogenous glucose production in the liver, while impaired glucose tolerance (2hPG hyperglycemia) is primarily associated with insulin resistance in skeletal muscle (29). Moreover, postprandial plasma glucose (PPG) levels were most strongly predictive of long-term glycemic control (reflected in glycated hemoglobin, HbA_1c_) in non-diabetic subjects, suggesting that PPG response may be more indicative than FPG for long-term glycemic maintenance (30). Our previous studies also point to the differences in the strength of association of FPG and 2hPG levels with TL (20, 21). In particular, no association was found between normal FPG and TL levels (20). Thereby, FPG or 2hPG levels might likely differently impact the link between TLs and MetS. In the longitudinal CARDIA Study (15), low HDL cholesterol level was associated with short TLs and the 10 years increase in waist circumference was associated with 10 years telomere attrition. Increased FPG level and MetS predicted greater 10 years decrease in leukocyte mitochondrial DNA copy number (alternative cellular aging marker) but not TLs decrease. The definition of MetS used in CARDIA Study, unlike IDF MetS definition, did not mandatory include an increase in waist circumference (15). It seems likely that variability of the components in the selection of MetS may affect the association of this metabolic category with markers of cellular aging.

The current IDF definition of MetS does not include the 2hPG data (1). IDF consensus, however, included 2hPG (OGTT) in the list of the additional metabolic criteria for MetS research [see Table 8 from (1)]. Therefore in this study we tested our hypothesis that impaired glucose tolerance can affect the relationship between MetS and TLs. Our study found that impaired glucose tolerance (2hPG) levels, but not FPG levels, affected the association between TLs and MetS, which may be also indicative for pathophysiological differences in these hyperglycemia categories. Based on the data obtained, it can be assumed that using 2hPG (but not FPG only) can provide an opportunity for a more accurate diagnostics of MetS and for evaluating the rate of aging (biological age) in patients with MetS. Further research, however, is needed to verify this assumption. The main limitation of our study was the small sample size. For this reason, the findings should be considered preliminary and suggestive, and need to be confirmed using larger samples in future research. One more limitation of our study is that we used cross-sectional design, which precludes causal inferences. So, longitudinal observations are needed to be further conducted in order to verify the findings of this study. Moreover, the relationship of 2hPG with TLs should be further investigated in the context of the liver, pancreas, and muscle aging and insulin resistance.

## Data Availability

The datasets generated for this study are available on request to the corresponding author.

## Author Contributions

MK and AV conceived the study design. VIK, VC, OK, and VS participated in data collection. DK, MK, VAK, VG, VMK, and AV participated in data analysis and interpretation. VAK and VG carried out the final statistical analysis. MK and AV drafted the manuscript and designed the figures. All authors approved the final version of the paper.

### Conflict of Interest Statement

The authors declare that the research was conducted in the absence of any commercial or financial relationships that could be construed as a potential conflict of interest.

## Supplementary Material

Figure S1 The architecture of the neural network MLP (with one hidden layer) model predicting the risk of reducing the length of telomeres: the triangles indicate the neurons of the input layer (signs MetS, Age, Gender, 2hPG); gray squares—neurons of the hidden layer (sigmoid activation function); white square—neuron of the output layer (sigmoid activation function).
