# Supplementary figures and images for: Additional Impact of Glucose Tolerance on Telomere Length in Persons With and Without Metabolic Syndrome in the Elderly Ukraine Population
Source: Front Endocrinol (Lausanne). 2019 Feb 28;10:128. doi: 10.3389/fendo.2019.00128 (PMC6404635; doi:10.3389/fendo.2019.00128)

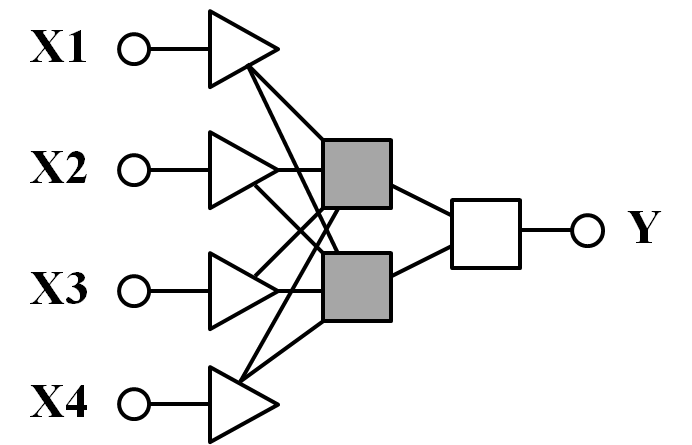

Supplement: Figure S1 — The architecture of the neural network MLP (with one hidden layer) model predicting the risk of reducing the length of telomeres: the triangles indicate the neurons of the input layer (signs MetS, Age, Gender, 2hPG); gray squares—neurons of the hidden layer (sigmoid activation function); white square—neuron of the output layer (sigmoid activation function). [file Image_1.TIF]
